# Nucleic acid amplification tests reduce delayed diagnosis and misdiagnosis of pulmonary tuberculosis

**DOI:** 10.1038/s41598-022-16319-8

**Published:** 2022-07-14

**Authors:** Jia-Yih Feng, Chou-Jui Lin, Jann-Yuan Wang, Shun-Tien Chien, Chih-Bin Lin, Wei-Chang Huang, Chih-Hsin Lee, Chin-Chung Shu, Ming-Chih Yu, Jen-Jyh Lee, Chen-Yuan Chiang

**Affiliations:** 1 Department of Chest Medicine, Taipei Veterans General Hospital, Taipei, Taiwan, ROC; 2 School of Medicine, National Yang Ming Chiao Tung University, Taipei, Taiwan, ROC; 3 Institute of Emergency and Critical Care Medicine, National Yang Ming Chiao Tung University, Taipei, Taiwan, ROC; 4 Tao-Yuan General Hospital, Ministry of Health and Welfare, Tao-Yuan, Taiwan, ROC; 5 Department of Internal Medicine, National Taiwan University Hospital, Taipei, Taiwan, ROC; 6 School of Medicine, College of Medicine, National Taiwan University, Taipei, Taiwan, ROC; 7 Chest Hospital, Ministry of Health and Welfare, Tainan, Taiwan, ROC; 8 Division of Chest Medicine, Department of Internal Medicine, Hualien Tzu Chi Hospital, Hualien, Taiwan, ROC; 9 School of Medicine, Tzu Chi University, Hualien, Taiwan, ROC; 10 Ph.D. Program in Translational Medicine, National Chung Hsing University, Taichung, Taiwan, ROC; 11 Division of Chest Medicine, Department of Internal Medicine, Taichung Veterans General Hospital, Taichung, Taiwan, ROC; 12 Department of Medical Technology, Jen-Teh Junior College of Medicine, Nursing and Management, Miaoli, Taiwan, ROC; 13 Master Program for Health Administration, Department of Industrial Engineering and Enterprise Information, Tunghai University, Taichung, Taiwan, ROC; 14 Division of Pulmonary Medicine, Department of Internal Medicine, Wan Fang Hospital, Taipei Medical University, Taipei, Taiwan, ROC; 15 Department of Internal Medicine, School of Medicine, College of Medicine, Taipei Medical University, 111 Hsin-Long Road, Section 3, Taipei 116, Taiwan, ROC; 16 School of Respiratory Therapy, College of Medicine, Taipei Medical University, Taipei, Taiwan, ROC; 17 International Union Against Tuberculosis and Lung Disease, Paris, France

**Keywords:** Infectious diseases, Microbiology

## Abstract

The clinical impact of nucleic acid amplification (NAA) tests on reducing delayed diagnosis and misdiagnosis of pulmonary TB (PTB) has rarely been investigated. PTB patients were classified into a frontline NAA group, an add-on NAA group, and a no NAA group. The outcomes of interest were the proportion of PTB case died before anti-TB treatment, the interval between sputum examination and initiation of treatment, and misdiagnosis of PTB. A total of 2192 PTB patients were enrolled, including 282 with frontline NAA, 717 with add-on NAA, and 1193 with no NAA tests. Patients with NAA tests had a lower death rate before treatment initiation compared to those without NAA tests (1.6% vs. 4.4%, p < 0.001) in all cases. Patients with frontline NAA compared to those with add-on NAA and those without NAA, had a shorter interval between sputum examination and treatment initiation in all cases (3 days vs. 6 days (p < 0.001), vs 18 days (p < 0.001)), and less misdiagnosis in smear-positive cases (1.8% vs. 5.6% (p = 0.039), vs 6.5% (p = 0.026)). In conclusion, NAA tests help prevent death before treatment initiation. Frontline NAA tests perform better than add-on NAA and no NAA in avoiding treatment delay in all cases, and misdiagnosis of PTB in smear-positive cases.

## Introduction

Pulmonary tuberculosis (TB) is an airborne transmitted infectious disease associated with high morbidity and mortality. In 2019, there were an estimated 10 million new TB cases and 1.2 million TB deaths globally^1^. Rapid diagnosis of TB with early initiation of anti-TB treatment is pivotal in the End TB strategy^2^. Delayed diagnosis of TB in critically ill patients may result in death before initiation of anti-TB treatment, which can be as high as 11% to 24% of the mortality cases in Taiwan^3,4^. Traditional microbiological tests for the diagnosis of TB include smear for acid-fast bacilli (AFB) and culture of *Mycobacterium tuberculosis* (MTB). The long turnaround time of mycobacterial cultures limits its application as a rapid diagnostic tool.

Nucleic acid amplification (NAA) tests for TB diagnosis have been commercially available for more than 20 years. Xpert MTB/RIF (Xpert) is a widely used NAA tests for diagnosis of TB and detection of rifampicin resistance. When used as an initial diagnostic test replacing smear microscopy, the sensitivity of Xpert was 98% for smear-positive pulmonary TB and 68% for smear-negative pulmonary TB, with a specificity of 99% for both^5,6^. NAA tests are more sensitive than smear microscopy and have shorter turnaround time than mycobacterial cultures^7^. However, studies on the impact of Xpert test on increased detection of TB cases reported conflicting findings^8,9^. In addition, whether implementation of NAA tests may reduce delayed diagnosis and misdiagnosis of TB has rarely been investigated^10,11^.

NAA tests have been recommended as an initial diagnostic test replacing smear microscopy^6,12^, or as an add-on test after smear microscopy^13,14^. When used as an initial diagnostic test, NAA tests can facilitate early diagnosis of TB. When used as an add-on test, NAA tests can improve diagnostic accuracy by excluding non-TB disorder, such as non-tuberculous mycobacterium (NTM). The latest Taiwan Guidelines for TB diagnosis and treatment recommend NAA tests as an initial diagnostic test, together with smear microscopy and culture^15^. However, NAA tests were frequently performed as an add-on test following a positive smear-microscopy result to exclude NTM, or after a negative smear-microscopy result if TB was suspected. TB is a notifiable disease in Taiwan and must be reported to the Taiwan Centers for Disease Control (CDC)^16^. However, patients may be advised by clinicians to stop anti-TB treatment after treatment initiation if a misdiagnosis is noted and diagnosis is changed to non-TB^17^. We hypothesized that the use of NAA tests may help to reduce delayed diagnosis, death before initiation of anti-TB treatment and misdiagnosis of TB. The aim of this retrospective cohort study was to evaluate the clinical impact of NAA tests on (1) death before initiation of anti-TB treatment, (2) delayed diagnosis of TB assessed by the interval from sputum examination to treatment initiation, and (3) misdiagnosis of TB.

## Results

### Patient characteristics

**Figure 1:**
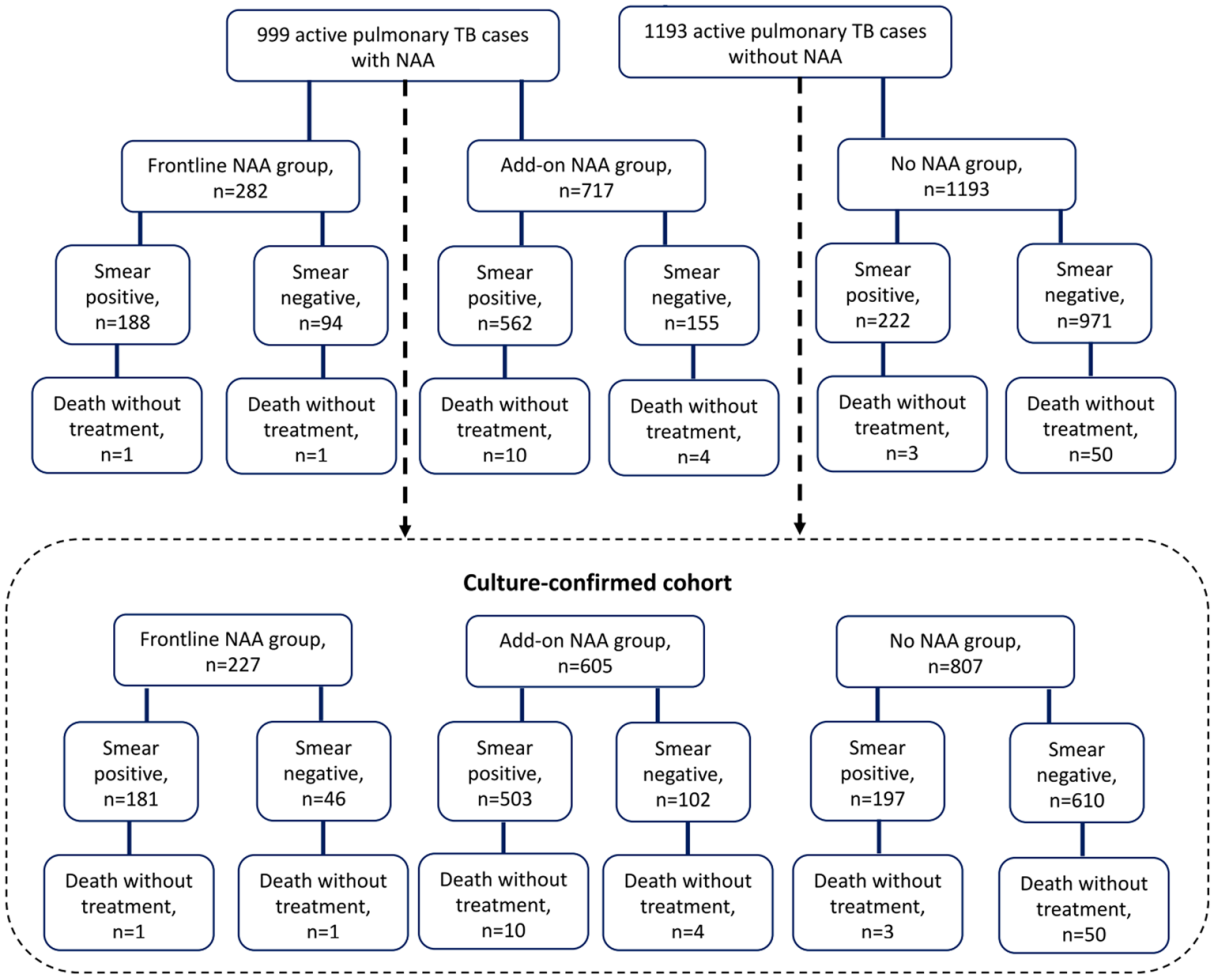
Study flow diagram and reasons for patient exclusion. *NAA* nucleic acid amplification.

Out of 4271 presumptive pulmonary TB patients with sputum NAA tests in 2017 and 2018 in the seven study hospitals, 1084 had been diagnosed with active pulmonary TB and reported to Taiwan CDC. Of the 1084 patients, 66 had missing information (date of smear microscopy, smear microscopy results, and date of treatment initiation) and 19 were younger than 20 years old. These 85 patients were excluded, and the remaining 999 patients were included for analysis. Of those, 282 (28.2%) had frontline NAA, and 717 (71.8%) had add-on NAA tests. There were 1193 adult pulmonary TB patients diagnosed in 2013–2014 in the study hospitals that did not conduct NAA tests; these patients were enrolled as the no NAA cohort for comparison (Fig. 1).

The demographic characteristics of enrolled pulmonary TB patients are shown in Table 1. Their mean age was 64.6 ± 19.6 years, 44.3% were sputum smear positive, and 74.8% were culture positive for MTB. Patients with NAA tests were more likely to be sputum smear positive (66.7% in frontline NAA group, 78.4% in add-on NAA group, 18.6% in no NAA group, p < 0.001) and culture positive for MTB (80.5% in frontline group, 84.4% in add-on group, 67.6% in no NAA group, p < 0.001). The median time from smear microscopy to NAA tests was 1 day (interquartile range [IQR] 0–2 days) in the frontline NAA group and 4 days (IQR 2–8 days) in the add-on NAA group.

**Table 1:** Characteristics of active pulmonary TB patients with and without NAA test.

	No NAA	With NAA		*p* value^a^	*p* value^b^	*p* value^c^
		Frontline NAA	Add-on NAA			
Case number	1193	282	717			
Age	64.1 (20.0)	65.6 (19.1)	65.0 (19.1)	0.170	0.268	0.650
Male sex	826 (69.2%)	197 (69.9%)	514 (71.7%)			
**Smear test results**				< 0.001	< 0.001	< 0.001
Positive	222 (18.6%)	188 (66.7%)	562 (78.4%)			
Negative	971 (81.4%)	94 (33.3%)	155 (21.6%)			
**Culture results**				< 0.001	< 0.001	0.139
MTB	807 (67.6%)	227 (80.5%)	605 (84.4%)			
NTM/no growth	386 (32.4%)	55 (19.5%)	102 (15.6%)			
Interval between smear test results and NAA results, median days (IQR)	–	1 (0–2)	4 (2–8)	–	–	< 0.001
**Enrolled hospital**				< 0.001	< 0.001	< 0.001
Hospital 1	308 (25.8%)	15 (5.3%)	108 (15.1%)			
Hospital 2	180 (15.1%)	59 (20.9%)	147 (20.5%)			
Hospital 3	122 (10.2%)	43 (15.2%)	95 (13.2%)			
Hospital 4	82 (6.9%)	46 (16.3%)	72 (10.0%)			
Hospital 5	192 (16.1%)	56 (19.9%)	106 (14.8%)			
Hospital 6	131 (11.0%)	30 (10.6%)	96 (13.4%)			
Hospital 7	178 (14.9%)	57 (18.8%)	69 (9.6%)			

Data are presented as n (%) unless otherwise mentioned.

*NAA* nucleic acid amplification, *TB* tuberculosis, *MTB Mycobacterium tuberculosis.*

^a^Comparison between patients with frontline NAA and no NAA test.

^b^Comparison between patients with add-on NAA and no NAA test.

^c^Comparison between patients with frontline and add-on NAA test.

### Impact of NAA tests on death before treatment

A total of 69 cases died without anti-TB treatment and the cause of death is shown in Table 2. Non-TB-related bacterial pneumonia with sepsis was the most common cause of death in these patients, followed by malignant diseases. The proportion of patients who died without anti-TB treatment was significantly lower among patients with NAA tests than in patients without NAA (1.6% vs. 4.4%, p < 0.001) (Table 3). In sup-group analysis, the difference remained significant in smear-negative patients (2.0% vs. 5.1%, p = 0.033), but not in smear-positive patients (1.5% vs. 1.4%, p = 0.368).

**Table 2:** Causes of death in PTB patients died before anti-TB treatment.

Causes of death	Case number (%)
Sepsis related to non-TB pulmonary infection	27 (39.1%)
Malignant diseases	18 (26.1%)
Sepsis related non-pulmonary infection	8 (11.6%)
Cardiovascular diseases	7 (10.1%)
Cerebrovascular diseases	5 (7.2%)
Active tuberculosis	3 (4.3%)
Others	7 (10.1%)

Data are presented as n (%).

**Table 3:** Death before anti-TB treatment among pulmonary TB patients with and without NAA test.

	No NAA	With NAA	*p* value^a^	NAA strategy		*p* value^b^	*p* value^c^	*p* value^d^
				Frontline NAA	Add-on NAA			
**Overall population**								
Case number	1193	999		282	717			
Death before treatment	53 (4.4%)	16 (1.6%)	< 0.001	2 (0.7%)	14 (2.0%)	0.003	0.004	0.159
**Smear-positive cohort**								
Case number	222	750		188	562			
Death before treatment	3 (1.4%)	11 (1.5%)	0.368	1 (0.5%)	10 (1.8%)	0.400	0.672	0.308
**Smear-negative cohort**								
Case number	971	249		94	155			
Death before treatment	50 (5.1%)	5 (2.0%)	0.033	1 (1.1%)	4 (2.6%)	0.077	0.165	0.653

Data are presented as n (%).

*NAA* nucleic acid amplification, *TB* tuberculosis.

^a^Comparison between patients with and without NAA tests.

^b^Comparison between patients with frontline NAA and no NAA test.

^c^Comparison between patients with add-on NAA and no NAA test.

^d^Comparison between patients with frontline and add-on NAA test.

### Impact of NAA on delayed treatment initiation

The median interval from sputum examination to treatment initiation is shown in Table 4. In smear-positive patients, the interval was significantly shorter in patients with frontline NAA but significantly longer in patients with add-on NAA, and in smear-negative patients the interval was also significantly shorter in patients with frontline NAA than that in patients without NAA. The median interval in patients with frontline NAA was 1 day shorter than that in patients without NAA in smear-positive cases (2 days vs. 3 days, p < 0.001) and 12 days shorter than that in patients without NAA in smear-negative cases (9 days vs. 21 days, p = 0.002). The median interval in patients with add-on NAA was 2 days longer than that in patients without NAA in smear-positive cases (5 days vs. 3 days, p < 0.001), and 3 days shorter than that in patients without NAA in smear-negative cases, although without significant differences (18 days vs. 21 days, p = 0.145).

**Table 4:** Interval between first sputum examination and anti-tuberculous treatment among pulmonary TB patients, stratified by NAA strategy.

	No NAA (A)	With NAA (B)	(B − A) Difference (95% CI)^a^	NAA strategy		(C − A) Difference (95% CI)^b^	(D − A) Difference (95% CI)^c^	(D − C) Difference (95% CI)^d^
				Front line NAA (C)	Add-on NAA (D)			
**Overall population**								
Case number	989	931		249	682			
Median (days)	18	5	− 10.5* (− 9.9 to − 12.0)	3	6	− 12.4* (− 15 to − 9.8)	− 9.7* (− 11.5 to − 8)	2.6* (0.5 to 4.8)
Interquartile range (days)	4–31	2–11		1–7	3–13			
**Smear-positive population**								
Case number	184	710		171	539			
Median (days)	3	4	0.1 (− 2.1 to 1.9)	2	5	− 3.4* (− 5.8 to − 1.0)	0.93 (− 1.3 to 3.2)	4.3* (2.3 to 6.4)
Interquartile range (days)	1–6.5	2–7		1–4	3–8			
**Smear negative population**								
Case numbers	805	221		78	143			
Median (days)	21	17	− 3.6* (− 0.7 to − 6.4)	9	18	− 5.7* (− 10.2 to − 1.2)	− 2.4 (− 5.8 to 1.0)	3.3 (− 1.9 to 8.5)
Interquartile range (days)	9–34	6–29		4–30	9–28			

Data are presented as n (%).

*NAA* nucleic acid amplification, *TB* tuberculosis.

^a^Comparison of mean differences between patients with and without NAA test.

^b^Comparison of mean differences between patients with frontline NAA and no NAA test.

^c^Comparison of mean differences between patients with add-on NAA and no NAA test.

^d^Comparison of mean differences between patients with frontline and add-on NAA test.

*p value < 0.05.

The proportion of patients that initiated anti-TB treatment within 28 days after the first sputum examination is shown in Supplementary Table S2. Smear-negative patients with NAA had significantly higher proportion of treatment initiation within day 7, day 14, day 21, and day 28, than those without NAA. When stratified by frontline NAA and add-on NAA, patients with frontline NAA had significantly higher proportions of treatment initiation within day 7 and day 14 than those without NAA among smear-positive patients, and significantly higher proportions of treatment initiation within day 7, day 14, and day 21 than those without NAA among smear-negative patients.

**Figure 2:**
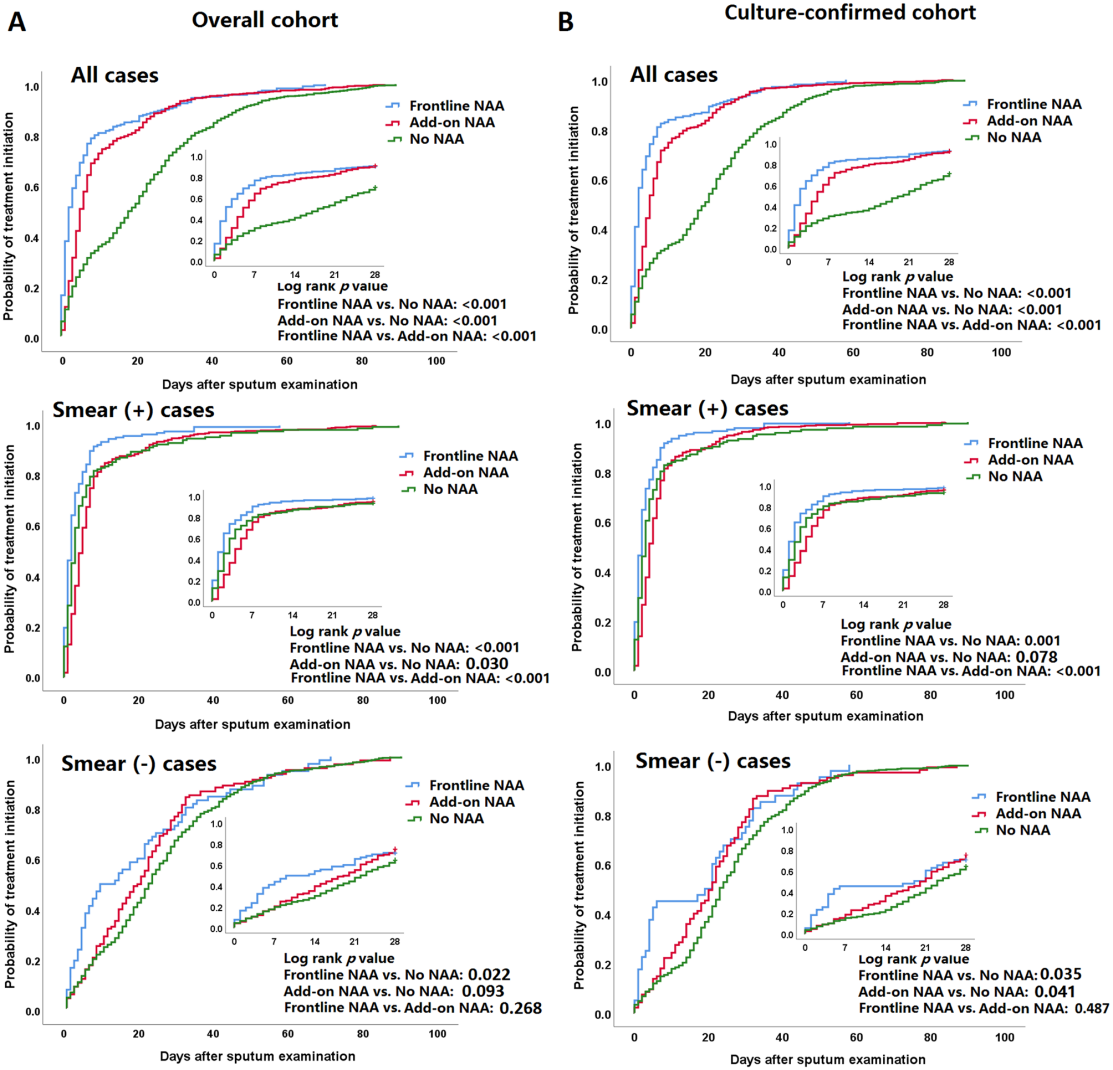
Kaplan–Meier analysis of time to initiation of anti-TB treatment in the (**A**) overall cohort, and (**B**) culture-confirmed cohort. *NAA* nucleic acid amplification.

The Kaplan–Meier curves demonstrating time to initiation of anti-TB treatment are shown in Fig. 2A. Patients with frontline NAA had significantly shorter interval from sputum examination to treatment initiation than those with add-on NAA and no NAA, irrespective of the sputum smear status. However, patients with add-on NAA had longer interval from sputum examination to treatment initiation than those without NAA in smear-positive cases.

### Impact of NAA tests on misdiagnosis of TB

Analysis of misdiagnosis is shown in Table 5. Patients with NAA tests had a significantly lower proportion of misdiagnosis than those without NAA (5.9% vs. 8.7%, p = 0.048), and the difference remained significant in the frontline NAA group (frontline NAA 4.8% vs. no NAA 8.7%, p = 0.043), but not significant in the add-on NAA group. In sub-group analysis, the proportion of misdiagnosis in frontline NAA groups was lower than that in the no NAA group in smear-positive patients (1.8% vs. 6.5%, p = 0.026), but not in smear-negative patients. The proportion of misdiagnosis was not significantly different between add-on NAA and no NAA cases. The proportion of misdiagnosis was significantly lower in patients with frontline NAA than in add-on NAA patients among smear positive cases (1.8% vs. 5.6%, p = 0.039). The proportions of NTM-related misdiagnosis in patients with frontline NAA, with add-on NAA, and no NAA were 33.3% (1/3), 70.0% (21/30), 50.0% (6/12) respectively in smear-positive population, and 22.2% (2/9), 23.1% (3/13), 21.6% (16/74) respectively in smear-negative population. Information regarding growth of NTM in misdiagnosis of TB is shown in Supplementary Table S1.

**Table 5:** Misdiagnosis in tuberculosis patients with and without NAA tests.

	No NAA	With NAA	*p* value^a^	NAA strategy		*p* value^b^	*p* value^c^	*p* value^d^
				Frontline NAA	Add-on NAA			
**Overall population**								
Case number	989	931		249	682			
Diagnosis change	86 (8.7%)	55 (5.9%)	0.048	12 (4.8%)	43 (6.3%)	0.043	0.072	0.395
**Smear positive population**								
Case number	184	710		171	539			
Diagnosis change	12 (6.5%)	33 (4.6%)	0.300	3 (1.8%)	30 (5.6%)	0.026	0.632	0.039
**Smear negative population**								
Case number	805	221		78	143			
Diagnosis change	74 (9.2%)	22 (10.0%)	0.730	9 (11.5%)	13 (9.1%)	0.498	0.969	0.561

Data are presented as n (%).

*NAA* nucleic acid amplification, *TB* tuberculosis.

^a^Comparison between patients with and without NAA tests.

^b^Comparison between patients with frontline NAA and no NAA tests.

^c^Comparison between patients with add-on NAA and no NAA tests.

^d^Comparison between patients with frontline and add-on NAA tests.

### Impact of NAA tests on culture-confirmed pulmonary TB cohort

To verify our findings, we restricted the analysis in 1639 cases with culture-confirmed pulmonary TB and found that patients with NAA tests were less likely to die without treatment initiation, especially in smear-negative cases (Supplementary Table S3).When compared to no NAA group, patients with frontline NAA had shorter interval from sputum examination to treatment initiation, and a higher proportion of patients had initiated anti-TB treatment within day 7, day 14, and day 21, in both smear positive and smear-negative patients (Supplementary Tables S4–S5, Fig. 2B), consistent with our findings in the overall cohort.

## Discussion

This multicentre retrospective cohort study conducted in Taiwan, a TB-endemic area with TB notification rate of 33 cases per 100,000 population in 2020^18^, evaluated the clinical impact of different approaches of NAA tests on delayed treatment initiation, death before anti-TB treatment, and misdiagnosis in patients with active pulmonary TB. Although our study showed that patients with NAA tests were associated with less death without anti-TB treatment, fewer misdiagnosis after the initiation of anti-TB treatment, and a shorter interval between sputum examinations and treatment initiation, the clinical benefits of NAA tests were heavily dependent on the sputum smear status and NAA strategies. Therefore, it is crucial to assess the contribution of NAA separately for smear-positive and smear-negative cases. Nearly 60% of enrolled patients with NAA tests had add-on NAA following positive smear microscopy, suggesting that NAA tests have been considerably underused to reduce delayed diagnosis of TB in smear-negative cases in Taiwan.

Death without anti-TB treatment has been previously assessed in a limited number of studies. In the US, 5.1% of TB was diagnosed at death, from 1985 to 1988, a percentage that was reduced to 2.1% in 2005^11,19^. In Taiwan, Chiang et al. reported that 4.9% of TB patients died without anti-TB treatment in 2003, and Wu et al. reported that 4.0% of TB cases reported to Taiwan CDC in 2006–2008 were notified after death^20,21^. To reduce the number of patients that die without anti-TB treatment, early diagnosis of TB is crucial. Our study showed trend of fewer cases of death without anti-TB treatment in patients with frontline NAA as compared to those with add-on NAA, although without statistical significance. Meanwhile, our data revealed that a majority of TB cases with death before anti-TB treatment died of non-TB causes. Therefore, the impact of frontline NAA in reducing TB mortality in Taiwan remains to be investigated.

In our study, frontline NAA performed better than no NAA in preventing delayed diagnosis of TB. One retrospective cohort study reported that NAA tests improved diagnostic accuracy, reduced diagnosis delay, and helped to avoid unnecessary contact investigations in presumptive TB^22^. Another nation-wide cohort study in Taiwan also reported that NAA tests in ICU facilitated early isolation and reduced nosocomial TB transmission in patients with critical illness^23^. In line with previous studies, we confirmed that NAA tests were associated with reduced misdiagnosis and earlier treatment initiation.

Clinical studies evaluating the clinical impact of NAA tests between smear-positive and smear-negative cohorts are limited. Marks et al. reported that the benefits of NAA tests in reducing respiratory isolation and invasive procedures are most significant in smear-positive/culture-negative cases^11^. Our findings indicated that the clinical impact of NAA tests was heavily associated with the sputum smear status. We found that NAA tests significantly reduced death without treatment initiation in smear-negative patients, but not in smear-positive patients. In contrast, the contribution of NAA tests in reducing misdiagnosis was evident in smear-positive cases with frontline NAA, but not in smear-negative cases. The application of NAA tests in smear-positive patients can reduce misdiagnosis by differentiating MTB from other organisms, especially NTM. Our findings indicated that smear-positive patients with frontline NAA had a lower proportion of NTM-related misdiagnosis as compared to those with add-on NAA and without NAA. Additionally, in smear-negative patients, NAA tests can facilitate early treatment initiation, because of the short turnaround time, compared to mycobacterial cultures. More interestingly, NAA tests significantly reduced the interval time from sputum examination to treatment initiation in smear-negative patients (17 days vs. 21 days), but also significantly prolonged the interval in smear-positive patients (4 days vs. 3 days), due to the fact that a high proportion of patients had add-on NAA, rather than frontline NAA. These findings suggest that the benefit of NAA tests would be different between smear-positive and smear-negative TB cases.

There are two different strategies when applying NAA tests for TB diagnosis, either frontline tests as initial diagnostic tool, or add-on tests used when smear microscopy results are available and additional diagnostic tests are necessary^13,14^. A retrospective cohort study in Italy found that using Xpert as an add-on test to smear microscopy increased the proportion of cases diagnosed by 21%^24^. When compared to frontline NAA without smear microscopy, add-on NAA tests following smear microscopy were associated with higher TB case detection rate in previous studies^25,26^. Analysis of the impact of different NAA test strategies on improving early diagnosis and decreasing misdiagnosis remains limited. In the present study, we found that, when compared to add-on NAA, patients with frontline NAA tests had a shorter interval between sputum examination and treatment initiation, and more patients initiated anti-TB treatment at day 7 and day 14, irrespective of the sputum smear status.

Misdiagnosis of TB has rarely been investigated before. Chiang et al. reported that 13.9% of the TB patients notified in Taipei had their TB diagnosis changed and were advised to stop anti-TB treatment^17^. Houben et al. reported that false-positive TB diagnoses had profound consequences for TB patients and prevention efforts, yet were usually overlooked in policy decision making^27^. Studies have reported that Xpert tests may reduce empirical treatment and overtreatment of TB^28–30^. In the present study, smear-positive patients with frontline NAA tests had fewer cases of misdiagnosis than those with add-on NAA. In this study, a significant proportion of smear-positive patients with add-on NAA initiated anti-TB treatment before the results of NAA tests were available, which may lead to misdiagnosis of TB. Our findings indicated that although both frontline and add-on NAA tests can improve the accuracy of TB diagnosis and accelerate treatment initiation, the clinical benefits of NAA tests are more prominent when used as frontline strategy. In areas with adequate medical resources, NAA tests should be used in conjunction with smear microscopy as initial tests, rather than add-on tests that are performed when the results of smear microscopy are available.

This study had several limitations. As a retrospective study with large sample size, some relevant clinical characteristics, such as the presence of cavitation, and underlying comorbidities, have not been included. In addition, the sputum smear status were not similar between patients with and without NAA test. However, we have performed separate subgroup analysis in smear-positive and smear-negative cases. Great variation of the proportions of cases with frontline NAA, add-on NAA, and no NAA was noted in each participating hospital, suggesting that application of NAA was highly heterogeneous in different hospitals. Finally, Taiwan is an area with low HIV incidence. The HIV status of enrolled cases was not included in our study design, but the general HIV rates in incident TB cases in Taiwan was only approximately 2.5–3%.

In conclusion, this multicentre, retrospective cohort study enrolled more than 2000 pulmonary TB patients and analysed the clinical benefit of NAA tests as frontline tests or add-on tests. We found that inclusion of NAA tests in diagnosis of pulmonary TB can improve diagnosis accuracy in smear-positive cases and accelerate treatment initiation in both smear-positive and smear-negative cases. The clinical benefits are more significant for frontline NAA than for add-on NAA tests. It highlighted the clinical benefits of NAA tests in diagnosis and treatment initiation of pulmonary TB, and the potential superiority of the frontline strategy compared to the add-on strategy. In TB endemic areas with adequate medical resources, inclusion of NAA tests as an initial diagnostic tool should be considered to optimise TB diagnostic accuracy and early treatment initiation.

## Methods

### Patients and settings

This was a retrospective cohort study performed in seven referral hospitals in Taiwan. Pulmonary presumptive TB patients, who had undergone NAA tests from January 2017 to December 2018 and were subsequently notified to Taiwan CDC with diagnosis of pulmonary TB, were enrolled. Pulmonary TB patients notified from January 2013 to December 2014 without NAA tests were included as a reference group (no NAA group), because NAA test had not yet been recommended as an initial diagnostic test by the Taiwan TB guidelines during that period. Exclusion criteria were: patients below 20 years old, active TB patients without pulmonary involvement, and active TB patients without sputum smear microscopy. Data regarding the age, sex, type of NAA tests, and detailed information of smear microscopy, including examination date and results, were collected from medical chart reviews. Patients with ≥ 1 positive AFB smear were defined as smear-positive cases, and patients never having a positive AFB smear were defined as smear-negative cases. The study protocol was approved by the Institutional Review Boards of all participating hospitals, and informed consent requirement was waived (IRB Nos: 2019-11-007BC, IRB_201702013RIND, N201903076, CE18193A, N201903076, IRB108-269-B).

### NAA tests and strategies

Among the seven participating hospitals, two used Roche Diagnostics (Grenzach-Whylen, Germany) Cobas Amplicor Mycobacterium tuberculosis assay, three used Xpert MTB/RIF (Cepheid, Sunnyvale, CA, USA), and two used both Xpert MTB/RIF and in-house TB PCR tests. Frontline NAA was defined as NAA test requested before or concomitantly with smear microscopy; add-on NAA was defined as NAA test requested after obtaining results from smear microscopy.

### TB outcome evaluation

Outcomes of interest in the present study included the number and proportion of TB patients that died without anti-TB treatment, the interval between sputum examination and initiation of anti-TB treatment (delayed diagnosis), and misdiagnosis of TB. Misdiagnosis of pulmonary TB was defined as notified pulmonary TB cases, who were advised by clinicians to stop anti-TB treatment before completion of the treatment course and were de-notified, due to TB misdiagnosis. Information regarding diagnosis change (misdiagnosis), date of death, and treatment initiation were retrieved from the TB registration database of Taiwan CDC. The date of first sputum examination, including smear microscopy, mycobacterium culture, and NAA tests, were acquired from chart reviews.

### Statistical analysis

Comparisons of demographic and clinical characteristics were performed using a chi-square test or Fisher’s exact test for categorical variables, and the t test or Mann–Whitney U test for continuous variables, depending on data distribution. Death without anti-TB treatment, misdiagnosis, and delayed diagnosis of TB were compared between groups with different NAA strategies accordingly. Analysis of delayed diagnosis and misdiagnosis included patients who had initiation of anti-TB treatment after sputum examinations; those who died before anti-TB treatment and those who had the initiation of treatment before sputum examination were excluded. Since the impact of NAA tests in diagnosis and treatment of tuberculosis may be different between smear-positive and smear-negative cases, analysis was stratified by smear microscopy results. A Kaplan–Meier analysis was conducted, and log-rank tests were performed to compare the time to treatment initiation among patients with different NAA strategies. Sensitivity analysis was performed in culture-confirmed cohorts to confirm the findings from the overall population. Statistical analyses were performed using SPSS version 20.0 software (IBM Corp., Armonk, NY, USA) and Stata Version 15 (Stata Corp LP, College Station, Texas, USA).

### Ethics approval and consent to participate

The study protocol was approved by the Institutional Review Boards of all participating hospitals, and informed consent requirement was waived (IRB Nos: Taipei Veterans General Hospital-2019-11-007BC, National Taiwan University Hospital—IRB_201702013RIND, Taipei Medical University—N201903076, Taichung Veterans General Hospital—CE18193A, Wan Fang Hospital—N201903076, Hualien Tzu Chi Hospital -IRB108-269-B).

The research was performed in accordance with the Declaration of Helsinki.

## Supplementary Information

Supplementary Tables.

## Data Availability

The datasets generated and/or analysed during the current study are not publicly available due to ethical restriction from participated hospitals, but are available from the corresponding author on reasonable request.

## Abbreviations

TB: Tuberculosis
AFB: Acid-fast bacilli
MTB: *Mycobacterium tuberculosis*
NAA: Nucleic acid amplification
CDC: Centers for Disease Control
ICU: Intensive care units
