# Supplementary material for: Nucleic acid amplification tests reduce delayed diagnosis and misdiagnosis of pulmonary tuberculosis
Source: Sci Rep. 2022 Jul 14;12:12064. doi: 10.1038/s41598-022-16319-8 (PMC9283405; doi:10.1038/s41598-022-16319-8)
Supplement: Supplementary file 1 — Supplementary Tables. [file 41598_2022_16319_MOESM1_ESM.doc]

**Supplementary Materials**

Title: Nucleic acid amplification tests reduce delayed diagnosis and misdiagnosis of pulmonary tuberculosis

Jia-Yih Feng^1,2,3^, Chou-Jui Lin^4^, Jann-Yuan Wang^5,6^, Shun-Tien Chien ^7^, Chih-Bin Lin ^8, 9^, Wei-Chang Huang ^10, 11, 12, 13^, Chih-Hsin Lee^14, 15^, Chin-Chung Shu^16^, Ming-Chih Yu ^14, 15,17^, Jen-Jyh Lee^8^, Chen-Yuan Chiang^14, 15, 18^

^1^Department of Chest Medicine, Taipei Veterans General Hospital, Taipei, Taiwan

^2^School of Medicine, National Yang Ming Chiao Tung University, Taipei, Taiwan

^3^Institute of Emergency and Critical Care Medicine, National Yang-Ming Chiao-Tung University, Taipei, Taiwan

^4^Tao-Yuan General Hospital, Ministry of Health and Welfare, Tao-Yuan, Taiwan

^5^Department of Internal Medicine, National Taiwan University Hospital, Taipei, Taiwan

^6^School of Medicine, College of Medicine, National Taiwan University, Taipei, Taiwan

^7^c, Ministry of Health and Welfare, Tainan, Taiwan

^8^Division of Chest Medicine, Department of Internal Medicine, Hualien Tzu Chi Hospital, Hualien, Taiwan

^9^School of Medicine, Tzu Chi University, Hualien, Taiwan

^10^Ph.D. Program in Translational Medicine, National Chung Hsing University, Taichung, Taiwan

^11^Division of Chest Medicine, Department of Internal Medicine, Taichung Veterans General Hospital, Taichung, Taiwan

^12^Department of Medical Technology, Jen-Teh Junior College of Medicine, Nursing and Management, Miaoli, Taiwan

^13^Master Program for Health Administration, Department of Industrial Engineering and Enterprise Information, Tunghai University, Taichung, Taiwan

^14^Division of Pulmonary Medicine, Department of Internal Medicine, Wan Fang Hospital, Taipei Medical University, Taipei, Taiwan

^15^Department of Internal Medicine, School of Medicine, College of Medicine, Taipei Medical University, Taipei, Taiwan

^16^Department of Internal Medicine, National Taiwan University Hospital, Taipei, Taiwan

^17^School of Respiratory Therapy, College of Medicine, Taipei Medical University, Taipei, Taiwan

^18^International Union against Tuberculosis and Lung Disease, Paris, France

**Table S1.** Cumulative proportions of patients with initiation of anti-tuberculous treatment within 28 days of sputum examination, stratified by smear results^a^

|  | No NAA | With NAA | *p* value^b^ | NAA strategy | | *p* value^c^ | *p* value^d^ | *p* value^e^ |
| --- | --- | --- | --- | --- | --- | --- | --- | --- |
|  |  |  |  | Front line NAA | Add-on NAA |  |  |  |
| Overall population |  |  |  |  |  |  |  |  |
| Case numbers | 989 | 931 |  | 249 | 682 |  |  |  |
| Day 7 | 331 (33.5%) | 617 (66.3%) | <0.001 | 187 (75.1%) | 430 (63.0%) | <0.001 | <0.001 | 0.001 |
| Day 14 | 435 (44.0%) | 728 (78.2%) | <0.001 | 204 (81.9%) | 524 (76.8%) | <0.001 | <0.001 | 0.096 |
| Day 21 | 576 (58.2%) | 778 (83.6%) | <0.001 | 216 (86.7%) | 562 (82.4%) | <0.001 | <0.001 | 0.114 |
| Day 28 | 706 (71.4%) | 837 (89.9%) | <0.001 | 223 (89.6%) | 614 (90.0%) | <0.001 | <0.001 | 0.833 |
| Smear positive population |  |  |  |  |  |  |  |  |
| Case numbers | 184 | 710 |  | 171 | 539 |  |  |  |
| Day 7 | 146 (79.3%) | 550 (77.5%) | 0.584 | 151 (88.3%) | 399 (74.0%) | 0.023 | 0.148 | <0.001 |
| Day 14 | 160 (87.0%) | 627 (88.3%) | 0.614 | 161 (94.2%) | 466 (86.5%) | 0.021 | 0.864 | 0.006 |
| Day 21 | 166 (90.2%) | 644 (90.7%) | 0.840 | 163 (95.3%) | 481 (89.2%) | 0.065 | 0.709 | 0.017 |
| Day 28 | 171 (92.9%) | 672 (94.6%) | 0.372 | 166 (97.1%) | 506 (93.9%) | 0.076 | 0.651 | 0.105 |
| Smear negative population |  |  |  |  |  |  |  |  |
| Case numbers | 805 | 221 |  | 78 | 143 |  |  |  |
| Day 7 | 185 (23.0%) | 67 (30.3%) | 0.023 | 36 (46.2%) | 31 (21.7%) | <0.001 | 0.732 | <0.001 |
| Day 14 | 275 (34.2%) | 101 (45.7%) | 0.002 | 43 (55.1%) | 58 (40.6%) | <0.001 | 0.140 | 0.038 |
| Day 21 | 410 (50.9%) | 134 (60.6%) | 0.010 | 53 (67.9%) | 81 (56.6%) | 0.004 | 0.208 | 0.100 |
| Day 28 | 535 (66.5%) | 165 (74.7%) | 0.020 | 57 (73.1%) | 108 (75.5%) | 0.235 | 0.032 | 0.689 |

^a^Data are presented as n (%)

^b^Comparison between patients with and without NAA tests

^c^Comparison between patients with frontline NAA and no NAA tests

^d^Comparison between patients with add-on NAA and no NAA tests

^e^Comparison between patients with frontline and add-on NAA tests

NAA, nucleic acid amplification; TB, tuberculosis

**Table S2**. Misdiagnosis related to growth of NTM among pulmonary TB patients, stratified by NAA strategy

|  | Overall | No NAA | Add-on NAA | Frontline NAA |
| --- | --- | --- | --- | --- |
| Smear positive population |  |  |  |  |
| Case number | 894 | 184 | 539 | 171 |
| Misdiagnosed cases | 45 | 12 | 30 | 3 |
| Culture positive for NTM | 28 (62.2%) | 6 (50%) | 21 (70%) | 1 (33.3%) |
| Culture negative for NTM | 17 (37.8%) | 6 (50%) | 9 (30%) | 2 (66.7%) |
| Smear negative population |  |  |  |  |
| Case number | 1026 | 805 | 143 | 78 |
| Misdiagnosed cases | 96 | 74 | 13 | 9 |
| Culture positive for NTM | 21 (22.9%) | 16 (21.6%) | 3 (23.1%) | 2 (22.2%) |
| Culture negative for NTM | 75 (78.1) | 58 (78.4%) | 10 (76.9%) | 7 (77.8%) |

**Table S3**. Death before anti-TB treatment initiation among culture-confirmed pulmonary TB patients with and without NAA test^a^

|  | No NAA | With NAA | *p* value^b^ | NAA strategy | | *p* value^c^ | *p* value^d^ | *p* value^e^ |
| --- | --- | --- | --- | --- | --- | --- | --- | --- |
|  |  |  |  | Front line NAA | Add-on NAA |  |  |  |
| Overall population |  |  |  |  |  |  |  |  |
| Case numbers | 807 | 832 |  | 227 | 605 |  |  |  |
| Death before treatment | 53 (6.6%) | 16 (1.9%) | <0.001 | 2 (0.9%) | 14 (2.3%) | 0.001 | <0.001 | 0.180 |
| Smear test positive |  |  |  |  |  |  |  |  |
| Case numbers | 197 | 684 |  | 181 | 503 |  |  |  |
| Death before treatment | 3 (1.5%) | 11 (1.6%) | 0.933 | 1 (0.6%) | 10 (2.0%) | 0.357 | 0.682 | 0.304 |
| Smear test negative |  |  |  |  |  |  |  |  |
| Case numbers | 610 | 148 |  | 46 | 102 |  |  |  |
| Death before treatment | 50 (8.2%) | 5 (3.4%) | 0.043 | 1 (2.2%) | 4 (3.9%) | 0.141 | 0.131 | 1.000 |

^a^Data are presented as n (%)

^b^Comparison between patients with and without NAA tests

^c^Comparison between patients with frontline NAA and no NAA tests

^d^Comparison between patients with add-on NAA and no NAA tests

^e^Comparison between patients with frontline and add-on NAA tests

NAA, nucleic acid amplification; TB, tuberculosis

**Table S4**. Interval between first sputum examination and anti-tuberculosis treatment initiation between culture-confirmed pulmonary TB patients with and without NAA^a^

|  | No NAA | With NAA | *Difference (95% CI)*^b^ | NAA strategy | | *Difference (95% CI)*^c^ | *Difference (95% CI)*^d^ | *Difference (95% CI)*^e^ |
| --- | --- | --- | --- | --- | --- | --- | --- | --- |
|  |  |  |  | Front line NAA | Add-on NAA |  |  |  |
| Overall population |  |  |  |  |  |  |  |  |
| Case numbers | 685 | 779 |  | 204 | 575 |  |  |  |
| Median | 20 | 4 | -12.4* (-10.8~-14.0) | 2 | 5 | -14.8* (-12.2~-17.4) | -11.6* (-9.8~-13.3) | 3.2* (1.2-5.2) |
| Interquartile range | 5-31 | 2-9 |  | 1-6 | 3-11 |  |  |  |
| Smear test positive cohort |  |  |  |  |  |  |  |  |
| Case numbers | 161 | 644 |  | 164 | 480 |  |  |  |
| Median | 3 | 4 | 1.0 (-0.9~-2.9) | 2 | 4 | -3.7* (-1.2~-6.2) | 0.1 (-2.0~2.2) | 3.6* (1.9~5.4) |
| Interquartile range | 1-6 | 2-7 |  | 1-4 | 2-7 |  |  |  |
| Smear test negative cohort |  |  |  |  |  |  |  |  |
| Case numbers | 524 | 135 |  | 40 | 95 |  |  |  |
| Median | 23 | 21 | -4.8* (-7.9~-1.6) | 20 | 21 | -7.4* (-12.7~-2.0) | -3.7* (-0.1~-7.3) | 3.6 (-2.5~9.8) |
| Interquartile range | 15-34 | 7-29 |  | 3-31 | 11-29 |  |  |  |

^a^Data are presented as n (%)

^b^Comparison of mean differences between patients with and without NAA test

^c^Comparison of mean differences between patients with frontline NAA and no NAA test

^d^Comparison of mean differences between patients with add-on NAA and no NAA test

^e^Comparison of mean differences between patients with frontline and add-on NAA test

NAA, nucleic acid amplification; TB, tuberculosis

**Table S5**. Cumulative proportions of culture-positive pulmonary TB patients with initiation of treatment within 28 days from sputum examination^a^

|  | No NAA | With NAA | *p* value^b^ | NAA strategy | | *p* value^c^ | *p* value^d^ | *p* value^e^ |
| --- | --- | --- | --- | --- | --- | --- | --- | --- |
|  |  |  |  | Front line NAA | Add-on NAA |  |  |  |
| Overall population |  |  |  |  |  |  |  |  |
| Case numbers | 685 | 779 |  | 204 | 575 |  |  |  |
| Day 7 | 206 (30.1%) | 549 (70.5%) | <0.001 | 165 (80.9%) | 384 (66.8%) | <0.001 | <0.001 | <0.001 |
| Day 14 | 256 (37.4%) | 631 (81.0%) | <0.001 | 174 (85.3%) | 457 (79.5%) | <0.001 | <0.001 | 0.069 |
| Day 21 | 370 (54.0%) | 669 (85.9%) | <0.001 | 182 (89.2%) | 487 (84.7%) | <0.001 | <0.001 | 0.111 |
| Day 28 | 485 (70.8%) | 717 (92.0%) | <0.001 | 188 (92.2%) | 529 (92.0%) | <0.001 | <0.001 | 0.943 |
| Smear test positive cohort |  |  |  |  |  |  |  |  |
| Case numbers | 161 | 644 |  | 164 | 480 |  |  |  |
| Day 7 | 129 (80.1%) | 514 (79.8%) | 0.930 | 147 (89.6%) | 367 (76.5%) | 0.017 | 0.336 | <0.001 |
| Day 14 | 139 (86.3%) | 579 (89.9%) | 0.192 | 156 (95.1%) | 423 (88.1%) | 0.006 | 0.550 | 0.010 |
| Day 21 | 145 (90.1%) | 595 (92.4%) | 0.332 | 158 (96.3%) | 437 (91.0%) | 0.024 | 0.710 | 0.027 |
| Day 28 | 150 (93.2%) | 618 (96.0%) | 0.130 | 160 (97.6%) | 458 (95.4%) | 0.059 | 0.264 | 0.228 |
| Smear test negative cohort |  |  |  |  |  |  |  |  |
| Case numbers | 524 | 135 |  | 40 | 95 |  |  |  |
| Day 7 | 77 (14.7%) | 35 (25.9%) | 0.002 | 18 (45.0%) | 17 (17.9%) | <0.001 | 0.424 | 0.001 |
| Day 14 | 117 (22.3%) | 52 (38.5%) | <0.001 | 18 (45.0%) | 34 (35.8%) | 0.001 | 0.005 | 0.315 |
| Day 21 | 225 (42.9%) | 74 (54.8%) | 0.013 | 24 (60.0%) | 50 (52.6%) | 0.036 | 0.080 | 0.432 |
| Day 28 | 335 (63.9%) | 99 (73.3%) | 0.040 | 28 (70.0%) | 71 (74.7%) | 0.440 | 0.041 | 0.570 |

^a^Data are presented as n (%)

^b^Comparison between patients with and without NAA tests

^c^Comparison between patients with frontline NAA and no NAA tests

^d^Comparison between patients with add-on NAA and no NAA tests

^e^Comparison between patients with frontline and add-on NAA tests

NAA, nucleic acid amplification; TB, tuberculosis
